# Toward targeted mental health support: the (in)congruence effect of resilience and social support among casino employees in Macau

**DOI:** 10.1186/s12889-026-27684-8

**Published:** 2026-05-12

**Authors:** Wei Cao, Anqi Situ, Wan U Lao, Heyong Shen, Zhehua Ying

**Affiliations:** 1 School of Humanities, Guangdong Peizheng College, Guangzhou, Guangdong China; 2 Faculty of Health and Wellness, City University of Macau, Macau, SAR 999078 China

**Keywords:** Conservation of Resources (COR) theory, Psychological resilience, Perceived social support, Macau’s gaming industry employees, Response surface analysis

## Abstract

**Objective:**

Based on Conservation of Resources (COR) theory, this study aimed to examine the (in)congruence effect of psychological resilience and social support on mental health outcomes (anxiety, depression, perceived stress) among Macau’s gaming industry employees, and to explore targeted public health implications for Macau’s mental health support system.

**Methods:**

A cross-sectional survey was conducted among in-service casino employees in Macau. Psychological resilience, perceived social support, anxiety, depression, and perceived stress were measured. Response surface analysis (RSA) was used to examine the congruence and incongruence effects of the two core mental resources.

**Results:**

Congruently high levels of resilience and social support were associated with the lowest levels of anxiety, depression, and perceived stress, indicating a cumulative advantage of different mental resources. Under incongruent conditions, the protective roles of the two resources diverged: social support was more critical for depression, whereas resilience was more strongly associated with lower perceived stress. Marital status significantly moderated the (in)congruence effect on anxiety.

**Conclusion:**

These findings highlight the complex (in)congruence effect of resilience and social support from a COR perspective. The results support the necessity of a mental health system that addresses the specific resource profiles of employees in the Macau gaming industry. The public health implications for the high-stress occupational populations are discussed.

**Supplementary Information:**

The online version contains supplementary material available at 10.1186/s12889-026-27684-8.

## Introduction

Occupational health psychology (OHP) is crucial to the wellbeing of individuals and organizations [1]. It is especially urgent in highly stressful occupational settings, such as the gaming industry [2]. The gaming industry in Macau—one of the world’s largest gambling hubs—contributes 63.1% of the local GDP at its peak in 2013, and plays a central role in economic and social development [3]. Unlike Las Vegas, where 63% of revenue is derived from non-gaming entertainment services, over 90% of Macau’s tourism revenue comes from casino gaming, and 60—70% of gaming revenue is generated by high-net-worth VIP baccarat business [4]. This unique business structure exposes gaming employees to distinctive stressors: extreme emotional labor for VIP clients, irregular shift work, and pervasive ethical concerns [5], thereby exposing employees to distinct mental health challenges.

Research has consistently shown that gaming industry employees are chronically exposed to moderate-to-high levels of occupational stress [5, 6], which is linked to emotional burnout, unhealthy lifestyle behaviors and mental health problems [7–9]. In a large-scale study of 1124 Macau casino dealers, over half of the participants exhibited clinically depressive symptoms, and a quarter met the threshold for anxiety symptoms [10].

Although gaming employees rely heavily on personal resources and social support to manage work-related challenges [11, 12], demanding workloads and irregular schedules could restrict family relationships [10, 13], while social isolation may ruin their support networks [14]. In addition, frequent rude behavior, verbal harassment, and even violence from gamblers [15], and persistent moral dilemmas inherent to the gaming context [12] also undermine their personal resilience. However, extant literature has predominantly focused on the adverse effects of these stressors, with limited attention to the protective resources that can buffer against these risks.

Conservation of resources (COR) theory is now one of the most widely cited frameworks in occupational health psychology [16]. The core idea proposes that resources are fundamental to maintaining positive mental health outcomes [17]. Based on this idea, gaming employees’ mental health challenges may stem from their chronically exhausted resources, making complementary resource investment particularly salient in this context [16]. A key concept in COR theory is that resources are assumed not to exist in isolation, but aggregate into “resource caravans” [16]. However, a longstanding debate in the COR literature concerns whether their effects are inherently context-dependent [18]. That is to say, different resources may have domain-specific effects, and the same resource might be protective in one situation but ineffective or even counterproductive in another [16]. In particular, certain resource combinations could have counterproductive effects under high-pressure conditions [19, 20]. The study of Macau’s gaming employees—who struggle in a chronically high-stress occupational setting with pervasive resource exhaustion—thus provides a valuable opportunity to address these unresolved theoretical debates.

Psychological resilience and social support are both well-established key protective resources for mental health [21, 22]. These two resources can exert additive effects, independently buffering against mental health risks [23, 24], or their interaction can further enhance the protective benefits [25, 26]. Previous research has demonstrated that a dual resource intervention targeting both social support and personal mental resources significantly reduced the depression in nurses [27]. These findings have confirmed the benefits of congruently high resource levels.

However, levels of resilience and perceived social support are not always congruent across individuals [28]. While research has reported that resilience and social support could buffer each other [29, 30], the combination of economic and psychological support could paradoxically reduce the resilience [31]. Previous studies have already explored programs to enhance the mental health of gaming industry employees from either a resilience [32] or social support [33] perspective, leaving the incongruent effect between two key resources largely untouched among the gaming employees in Macau. Traditional analytical approaches (e.g., hierarchical regression) cannot distinguish between the effects of congruently versus incongruent resource profiles. Recently, research applied response surface analysis (RSA) to adolescents and found congruently high resources predicted better mental health, with social support being most critical for stress [34]. The current study extends this inspiring work in several ways: (1) examining this (in)congruence effect in a chronic high-pressure occupational setting with special stressors, and identifying the relative importance of resilience and social support to different mental health problems (anxiety, depression and stress). (2) conducting an exploratory moderation analysis using the block variable method [35] to address the effect of different covariates.

**Fig. 1:**
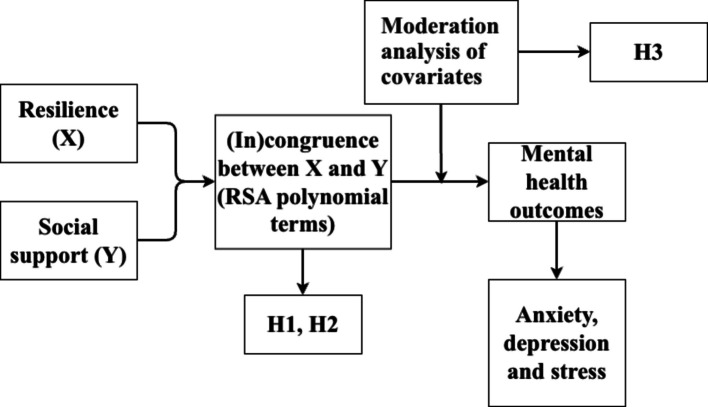
Conceptual framework of the current study. The model depicted the (in)congruence effect of psychological resilience (X) and social support (Y) on three mental health outcomes (Z: anxiety, depression, perceived stress), which directly tested the hypotheses H1 and H2. Covariates including marital status, position, and work shift were tested as moderators of the (in)congruence effect (H3)

Based on COR theory and prior evidence, we propose the hypotheses: H1: Congruently high levels of resilience and social support will be associated with lower anxiety, depression, and perceived stress. Based on the idea of resource caravan and prior research, the congruently high levels of both resources may show additive effects on buffering the mental health challenges. H2: According to the context-dependent nature of resources, under incongruent situation, the protective effects of both resources will diverge. Though the current study proposes that social support maybe critical to the perceived stress in accordance to previous research [34], the effect of different resources profiles would be tested under this special occupational contexts. Previous research has indicated several confounding factors that may impact the relationships between the two resources and mental health issues, like age, gender, marital status, length of employment and positions [13], the moderation effect was unexplored yet. Thus the current study would conduct an exploratory analysis to test the last hypothesis: H3: several covariates would moderate the effect between resources and mental health challenges. Identifying such resources-mental health profiles could offer valuable insight for identifying high-risk employees and providing reasonable mental health interventions. The corresponding research model is shown in Fig. 1.

## Methods

### Participants

The study protocol and survey instruments were approved by the Human Research Ethics Committee of the City University of Macau (No. 2024-RE-45). Participants were full-time gaming employees aged 21 years or older. Recruitment was conducted through two channels: 96% via the Gaming Industry Employees’ Home (representing six major local gaming operators), and 4% through research assistants distributing survey links in casino staff rest areas. Part-time, temporary, and non-gaming personnel were excluded.

All participants provided electronic informed consent and completed an anonymous online questionnaire. The information sheet outlined the study purpose, data anonymity, voluntary nature of participation, and the right to withdraw without consequences. After finishing the questionnaire, participants could optionally enter a prize draw for supermarket or McDonald’s vouchers (two of MOP 500, three of MOP 200, fifteen of MOP 100). Of 747 initial responses, 48 were excluded based on predefined criteria: completion time under 180 s (n = 30), demographic inconsistencies (n = 7), non-gaming or missing job titles (n = 5), and consecutive identical responses (n = 12). A total of 703 valid responses were retained, yielding an effective response rate of 94.11%.

### Measures

Anxiety and depression were assessed by the 7-item Generalized Anxiety Disorder scale (GAD-7) and the 9-item Patient Health Questionnaire (PHQ-9), respectively. Both are validated self-report instruments aligned with DSM-5 diagnostic criteria [36, 37], using a 4-point Likert scale (0 = not at all to 3 = nearly every day). Total GAD-7 scores range from 0–21 (minimal: 0–4, mild: 5–9, moderate: 10–14, severe: 15–21), and PHQ-9 scores range from 0–27 (minimal: 0–4, mild: 5–9, moderate: 10–14, moderately severe: 15–19, severe: 20–27). Sample item: GAD-7 (“Feeling nervous, anxious, or on edge”),PHQ-9 (“Little interest or pleasure in doing things”). Both scales showed excellent internal reliability (GAD-7: *Cronbach’s α* = 0.951; PHQ-9: *Cronbach’s α* = 0.918).

Perceived stress was measured using the 14-item Chinese Perceived Stress Scale [38]. Items are rated on a 5-point Likert scale (1 = never to 5 = always), with total scores ranging from 14–70 (low: 14–28, moderate: 29–42, high: 43–56, very high: 57–70). Sample item: “In the last month, how often have you felt unable to control important things in your life?” Internal reliability was good in the current study (*Cronbach’s α* = 0.804).

Psychological resilience was assessed via the 10-item Chinese version of the Connor-Davidson Resilience Scale (CD-RISC-10, [39]). Items are rated on a 5-point Likert scale (0 = never true to 4 = nearly always true), with total scores ranging from 0–40; higher scores indicate greater resilience. Sample item: “Being able to adapt to change when changes occur”. Internal reliability was excellent (*Cronbach’s α* = 0.956).

Perceived social support was measured with the Chinese version of the Perceived Social Support Scale (PSSS), which includes three subscales: family, friends, and significant others [40]. Items are rated on a 7-point Likert scale (1 = very strongly disagree to 7 = very strongly agree), with total scores ranging from 12–84. Sample item: “My family really tries to help me”. Internal reliability was excellent (*Cronbach’s α* = 0.960).

The present study also included the following covariates on the basis of the previous research [2, 10, 12]: sex assigned at birth (female, male), age group (21–35, 36–45, 46–55, 56–65 years), working experience (0–5, 5–10, 10–15, 15–20, 20 years and above), educational level (junior secondary education or below, senior secondary education, tertiary education, undergraduate level or higher), marital status (married, not married, separated/divorced), job position (services and sales workers, managers and administrators, technicians and associate professionals, clerks [excluding croupiers], croupiers), monthly income in MOP (0–20,000, 20,001–30,000, 30,001–40,000, 40,001 and above), and work schedule (shift work, fixed-schedule work, irregular shift).

### Data analysis

To examine the hypothesized congruence effects, the current study utilized the response surface analysis (RSA) method, which integrated a second-degree polynomial regression model with response surface methodology [41]. For each mental health outcome (anxiety, depression, and perceived stress), a polynomial regression model was fitted where the predictor variables were psychological resilience (X), social support (Y), their squared terms, their interaction term, and additive covariates (C).

$$\begin{aligned}Z&=b0+b1\times X+b2\times Y\\&+b3\times {X}^{2}+b4\times {Y}^{2}+b5\times X\times Y\\&+\sum (b{6}_{k}\times {C}_{k})+\varepsilon\ (K=1, 2, 3\cdots \cdots )\end{aligned}$$

From the resulting polynomial regression coefficients (*b1–b5*), five key response surface parameters (*a1–a5*) were computed. These parameters quantify the slopes and curvatures along the lines of congruence (LOC) and incongruence (LOIC), thereby directly testing how the congruence and incongruence of the two resources are related to employee mental health. These two predictors were standardized into z-scores to eliminate scale differences and reduce multicollinearity in the polynomial regression model [42].

To test the moderation effects of different covariates, the block variable approach was utilized [35]. A block variable for each mental health outcome was first computed with polynomial regression coefficients (*b1–b5*) that represent the joint effect (in)congruence.

$$\begin{aligned}block\ variable&=b1\times X+b2\times Y\\&+b3\times {X}^{2}+b4\times {Y}^{2}\\&+b5\times X\times Y\end{aligned}$$

The different covariates were subsequently tested as moderators between the block variable and the corresponding mental health outcomes.

All the analyses were conducted via the RSA package (0.10.8) and bruceR package [43] in R (4.5.1).

### Sensitivity analysis

Sensitivity analyses were conducted to examine the robustness of the findings. First, model stability was assessed by systematically comparing results with and without adjustment for covariates. Second, the validity of the model specification was tested by evaluating a sequence of nested models, including linear-only terms, linear terms with interaction components, and the full model [34]. Additionally, multicollinearity was evaluated through variance inflation factors *(VIFs*).

## Results

### Preliminary results

Table 1 presents the demographic characteristics of the study sample. Among the recruited participants, 28.31% and 45.66% showed mild level or above anxiety and depression, respectively. Moreover, 41.39% of the participants reported high or extremely high levels of perceived stress.

**Table 1:** Demographic characteristics of the study sample

**Variables**	*N* (%)	**Variables**	*N* (%)
Sex Assigned at Birth		Income (MOP)	
female	446(63.44)	0–20000	180(25.60)
male	257(36.56)	20001–30000	399(56.76)
Age		30001–40000	95(13.51)
21–35	96(13.66)	40,001 and above	29(4.13)
36–45	384(54.62)	Work Shift	
46–55	189(26.88)	Shift work	584(83.07)
56–65	34(4.84)	Fixed-schedule work	97(13.80)
Working Experience		Irregular shift	22(3.13)
0–5 years	67(9.53)	Anxiety	
5–10 years	129(18.35)	Minimal	292(41.54)
10–15 years	242(34.42)	Mild	212(30.16)
15–20 years	199(28.31)	Moderate	125(17.78)
20 years and above	66(9.39)	Severe	74(10.53)
Educational Levels		Depression	
Junior Secondary Education or Below	148(21.05)	Minimal	206(29.30)
Senior Secondary Education	298(42.39)	Mild	176(25.04)
Tertiary Education	113(16.07)	Moderate	189(26.88)
Undergraduate Level or Higher	144(20.48)	Moderately Severe	84(11.95)
Marital Status		Severe	48(6.83)
Married	576(81.93)	Perceived Stress	
Not married	60(8.53)	Low	54(7.68)
Separated/Divorced	67(9.53)	Mild	358(50.92)
Position		High	272(38.69)
Services and Sales Workers	42(5.97)	Extremely High	19(2.70)
Managers and Administrators	192(27.31)		
Technicians and Associate Professionals	54(7.68)		
Clerks (without croupiers)	55(7.82)		
Croupiers	360(51.12)		

Table 2 presents the mean scores, standard deviations, and correlations for all the predictors and outcomes. All the variables were significantly correlated with one another. Notably, both resilience and social support were significantly and negatively correlated with mental health outcomes.

**Table 2:** Means, standard deviations and Pearson’s correlations of each variable

Variable	Mean	SD	1	2	3	4	5
1. Depression	9.04	6.35	1				
2. Anxiety	6.69	5.72	0.849^***^	1			
3. Perceived stress	27.16	7.92	0.605^***^	0.650^***^	1		
4. Resilience	20.21	9.30	−0.304^***^	−0.318^***^	−0.623^***^	1	
5. Social support	53.94	15.51	−0.381^***^	−0.367^***^	−0.453^***^	0.503^***^	1

^*^ *p* < 0.05, ^**^ p < 0.01, ^***^ *p* < 0.001

### RSA for psychological resilience and social support

**Fig. 2:**
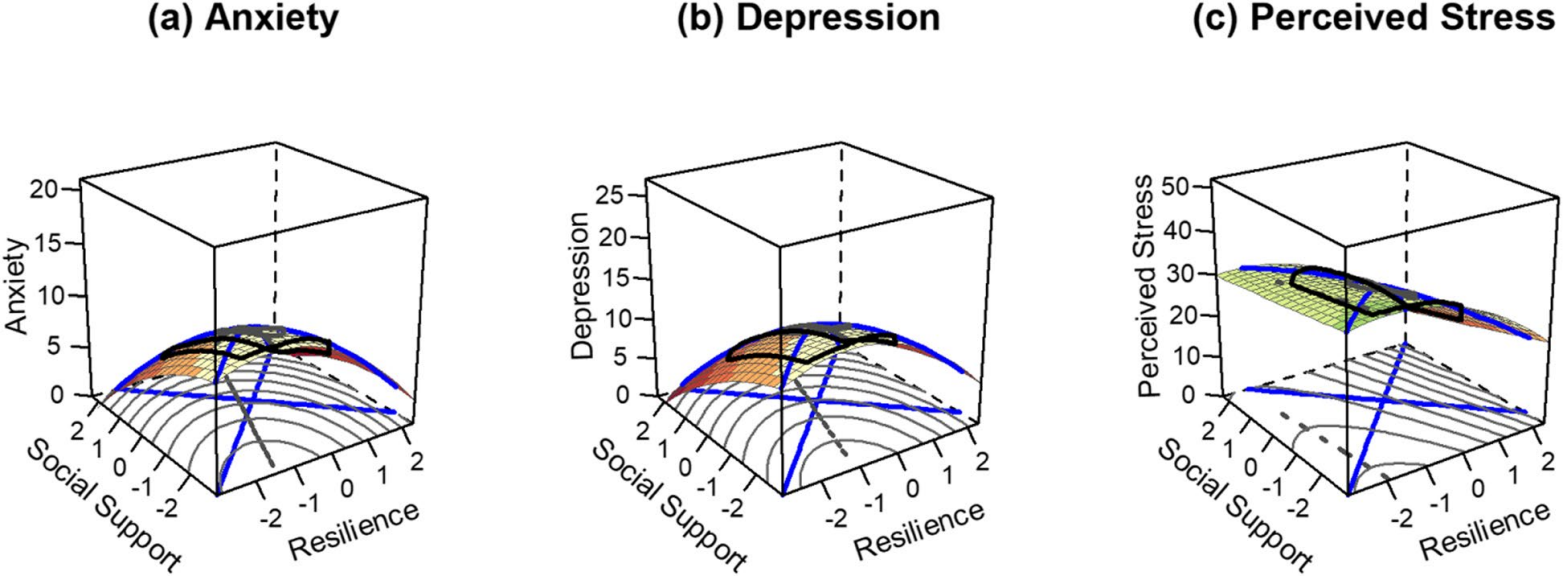
Plots of RSA results for resilience and social support related to mental health. **a** Anxiety, **b** depression, and **c** perceived stress. Resilience and social support were normalized before analysis and visualization

Table 3 presents the results of the response surface parameters via RSA. Furthermore, the plots in Fig. 2a-c provide a visual illustration of the results for the three mental health outcomes.

**Table 3:** Results of the response surface parameters

	Anxiety (SE)	Depression (SE)	Perceived stress (SE)
b0	5.922^***^ (1.789)	10.537^***^ (2.229)	31.778^***^ (2.121)
*b1* ~ resilience	−1.028^***^ (0.252)	−0.911^***^ (0.270)	−4.074^***^ (0.282)
*b2* ~ social support	−1.593^***^ (0.258)	−1.914^***^ (0.278)	−1.537^***^ (0.293)
*b3* ~ resilience^2^	−0.574^**^ (0.201)	−0.703^**^ (0.217)	−0.947^***^ (0.236)
*b4* ~ social support^2^	−0.281 (0.243)	−0.281 (0.234)	0.030 (0.239)
*b5* ~ resilience^*^social support	0.265 (0.262)	0.290 (0.257)	−0.184 (0.274)
*a1*	−2.620^***^ (0.286)	−2.824^***^ (0.322)	−5.611^***^ (0.328)
*a2*	−0.590^*^ (0.272)	−0.694^*^ (0.315)	−1.100^***^ (0.290)
*a3*	0.565 (0.423)	1.003^*^ (0.443)	−2.537^***^ (0.473)
*a4*	−1.120^*^ (0.457)	−1.273^**^ (0.425)	−0.733 (0.473)
*a5*	−0.294 (0.355)	−0.422 (0.360)	−0.977^*^ (0.382)

^*^ *p* < 0.05, ^**^ p < 0.01, ^***^ *p* < 0.001

Along the line of congruence (LOC), the slope (*a1*) was significantly negative for all three mental health outcomes (anxiety: *a1* = − 2.620, *p* < 0.001; depression: *a1* = − 2.824, *p* < 0.001; perceived stress: *a1* = − 5.611, *p* < 0.001), indicating that higher levels of congruent psychological resilience and social support were associated with better mental health. The relationships were nonlinear for anxiety (*a2* = − 0.590, *p* < 0.050), depression (*a2* = − 0.694, *p* < 0.050), and perceived stress (*a2* = − 1.100, *p* < 0.001), indicating an accelerating decrease in anxiety, depression, and perceived stress along the synchronized increase in resilience and social support.

The slope of the incongruence line (*a3*) was significantly positive for depression (*a3* = 1.003, *p* < 0.050), suggesting that greater social support coupled with lower resilience was associated with lower symptom levels. The curvature (*a4*) was significantly negative for anxiety (*a4* = − 1.120, *p* < 0.05) and depression (*a4* = − 1.273, *p* < 0.010). For perceived stress, the slope of the incongruence line was significantly negative (*a3* = − 2.537, *p* < 0.001), whereas the curvature was not significant (*a4* = − 0.733, *p* > 0.050).

The test of symmetry (*a5*) was non-significant for anxiety and depression but significant for perceived stress (*a5* = − 0.977, *p* < 0.050), indicating asymmetry in the latter’s response surface.

### Moderator analysis and subgroup RSA

**Fig. 3:**
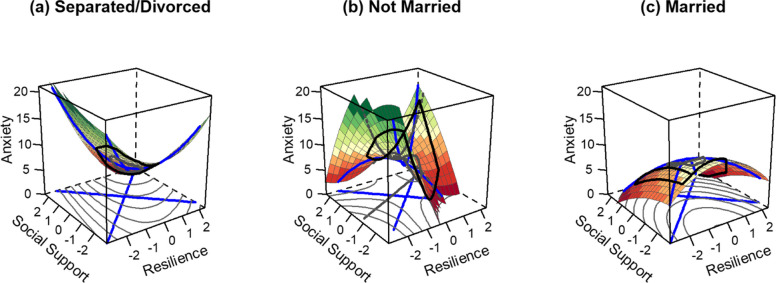
Plots of subgroup RSA results of resilience and social support for anxiety by marital status. **a** separated/divorced, **b** not married, **c** married. Resilience and social support were normalized before analysis and visualization

The exploratory moderator analysis revealed a significant moderation effect of marital status (*F* = 4.599, *p* = 0.010) on the relationship between (in)congruence and anxiety. Table 4 shows the subsequent subgroup RSA results. For participants who were separated/divorced and married, the slope of LOC was significantly negative (separated/divorced: *a1* = − 3.735, *p* < 0.001; married: *a1* = − 2.696, *p* < 0.001), and this relationship was linear for separated/divorced participants (*a2* = 0.189, *p* > 0.05) and nonlinear for married participants (*a2* = 0.993, *p* < 0.001). The slope of the LOIC (*a3*) was significantly positive for married participants (*a3* = 1.214, *p* < 0.01), and this relationship was nonlinear (*a4* = −1.029, *p* < 0.05). In contrast, single participants showed no significant relationship along the LOC (*a1* = −0.744,* p* > 0.05) but significant upward curvature (*a2* = 2.335,* p* < 0.001) (Fig. 3).

**Table 4:** Results of the subgroup RSA on anxiety by marriage status

	Separated/Divorced	Not married	Married
b0	5.979^***^ (0.862)	6.658^***^ (0.890)	7.660^***^ (0.288)
*b1* ~ resilience	−2.472^**^ (0.918)	−1.719 (0.948)	−0.741^**^ (0.260)
*b2* ~ social support	−1.263 (0.746)	0.975 (1.457)	−1.955^***^ (0.256)
*b3* ~ resilience^2^	0.946 (0.713)	0.879 (0.777)	−0.765^***^ (0.198)
*b4* ~ social support^2^	0.213 (0.712)	−0.735 (0.997)	−0.246 (0.247)
*b5* ~ resilience^*^social support	−0.970 (0.978)	2.191^*^ (1.013)	0.018 (0.251)
*a1*	−3.735^***^ (0.693)	−0.744 (1.029)	−2.696^***^ (0.293)
*a2*	0.189 (0.532)	2.335^***^ (0.554)	−0.993^***^ (0.279)
*a3*	−1.209 (1.523)	−2.694 (2.232)	1.214^**^ (0.425)
*a4*	2.129 (1.937)	−2.047 (2.087)	−1.029^*^ (0.431)
*a5*	0.733 (0.986)	1.614 (1.375)	−0.519 (0.363)

^*^ *p* < 0.05, ^**^ p < 0.01, ^***^ *p* < 0.001

### Sensitivity analysis

The RSA analysis was conducted by controlling for covariates, and the results revealed that position (*β* = 0.400, *p* < 0.001) and work shift (*β* = 1.187, *p* < 0.050) were significant covariates for anxiety, work shift (*β* = 1.922, *p* < 0.010) was significant for depression, and marital status (*β* = −1.109, *p* < 0.010) was significant covariate for perceived stress. Nevertheless, the RSA results remained stable with and without covariates, confirming the robustness of the analysis (Table S2 in the supplementary material).

Models with sequentially added terms—Model 1 (additive: first-order only), Model 2 (IA: first-order plus interaction), and Model 3 (full RSA: all second-order terms)—all showed low collinearity (*VIF* < 5). The AIC indicated the best fit for Model 3, whereas the BIC slightly favored Model 1 (Table S3 in the supplementary material). The full RSA model was selected based on theoretical considerations: it enables comprehensive examination of linear, quadratic, and interactive effects and exploration of the (in)congruence pSattern between the two resources.

## Discussion

While psychological resilience and social support are both well established as protective resources for mental health, the current study is among the first to apply response surface analysis (RSA) to explore the impact of (in)congruence between these two resources on the mental health of Macau’s gaming industry employees, and to address the context-dependent nature of resource effects in this occupational setting. By employing response surface analysis (RSA), this study provides new insight into the mental health support system of this workforce, which is vital to the region’s economy and public health. The high prevalence of mental health challenges—28.31% for anxiety, 45.66% for depression, and 41.39% for high to extreme perceived stress—underscores a pressing occupational and public health issue. Moreover, the current results indicate that symptoms of anxiety, depression and perceived stress decrease nonlinearly with increasing congruent psychological resilience and social support. Additionally, some new results revealed in the analysis of incongruence between resilience and social support. The level of perceived stress decreased with increased psychological resilience, which appeared to be the predominant protective factor. Moreover, higher social support coupled with lower resilience was associated with lower depression scores, suggesting that social support was more important in depression. These findings highlight the complex interplay between resilience and social support, underscoring the need for interventions based on the specific resource profiles of employees within the gaming industry.

Consistent with Hypothesis 1, the current results demonstrated that congruently high levels of psychological resilience and social support were associated with significantly lower anxiety, depression, and perceived stress among Macau gaming employees, which supported the idea of the resource caravan proposition in COR theory [16]. The RSA added new insights on this point by revealing that congruently higher levels of psychological resilience and social support are associated with disproportionately greater improvements in mental health outcomes, suggesting a cumulative advantage of resources for mental health [44]. This finding provided direct empirical support for COR theory’s resource gain spiral proposition [16]. In the chronically high-stress environment of Macau’s casinos, the initial resource accumulation seemed to create a self-reinforcing cycle of protective effect. While COR theory originally emphasized the primacy of resource loss spirals over gain spirals, the current results were aligned with recent evidence showing that key resource gains can have particularly protective effects in chronically stressed populations [45]. These findings highlight the importance of investigating resource gain spirals in high-stress occupational contexts. Critically, this result may imply that even employees with already high levels of resilience and social support can benefit substantially from further targeted resource enhancement, rather than interventions being limited exclusively to low-resource individuals.

Consistent with Hypothesis 2, the current results revealed divergent protective effects of resilience and social support under conditions of resource incongruence, and supported the context-dependent nature of resource effects [16, 18]. However, in contrast to the results of Zhu et al. [34], the current study found that resilience plays a crucial positive role in Macau’s gaming employees’ perceived stress, while social support mainly buffered against depression. This significant difference may suggest a hierarchical reorganization of the resource caravan under an occupation with chronically high-pressure.

Psychological resilience has been well established as a main resource against perceived stress [46, 47]. People with higher resilience possess greater available coping resources, which reduces their perception of stressors as threatening and thus lowers perceived stress [48, 49]. However, research has shown that resilience seems to buffer the cognitive perception of stress rather than the underlying physiological responses [50]. It is these underlying physiological responses that are related to anxiety and depression in a stressful context [51]. Especially in occupational settings characterized by chronically high stress, resilience may deplete emotional energy [52] and drain already limited resources [20], thereby paradoxically exacerbating the very physiological stress responses it is intended to buffer. In contrast, depression is more closely linked to interpersonal resource loss and chronic physiological activation [51, 53], and social support—a core external resource—has been shown to provide stronger protective effects [54, 55]. This divergence in underlying mechanisms also explains why prior longitudinal studies have reported an unstable relationship between resilience and depression [56].

Regarding the exploratory moderation analysis, current results partially supported Hypothesis 3: only marital status significantly moderated the (in)congruence effect on anxiety. This differential moderation effect can be explained by the distinct meaning profiles of anxiety and depression, as anxiety is associated primarily with a focus on one’s internal world [53]. For married employees, marital status could constitute the most salient and consistent source of social support and profoundly shape the family resources. For separated/divorced participants, who benefited the most from high congruence of resources, and single participants, who showed no significant (in)congruence effect, their social support networks were heterogeneous, the family resource profile was inadequate for their overall social support context and even showed negative effects [57]. In contrast, depression is characterized by a dual focus on both personal and interpersonal resources [53], and individuals with depression actively monitor interpersonal resources across multiple domains, not just marital relationships.

These findings support the idea that resource effects are inherently context-dependent [58], and this context dependence may be particularly pronounced in Macau’s gaming industry, where employees face chronically high levels of stress with special stressors like emotional labor, irregular shift work, and pervasive ethical dilemmas. This unique context may reverse the positive effect of social support on stress [34], and requires targeted mental health interventions in the gaming industry, highlighting the need to address resource profiles rather than simply increasing the resource levels.

## Conclusions and implications

This study used response surface analysis to examine the effects of (in)congruence between resilience and social support on the mental health of Macau gaming employees. The results demonstrated that congruently high levels of both resources are associated with the best mental health outcomes. In conditions of incongruence, the protective functions of the two resources diverged: social support emerged as more critical for depression, whereas resilience played a more weighted role in reducing perceived stress. Marital status was found to moderate the (in)congruence effect on anxiety. These findings highlight the need for further considerations in mental health interventions.

First, the mental health support system should systematically consider the (in)congruence of resources. Employees with high resilience but low social support—at elevated depression risk despite adequate stress coping—should receive social support programs. Those with high social support but low resilience need to focus on resilience training to reduce perceived stress. This addresses the limitations of single-resource interventions [27].

Second, interventions should expand from remedial to preventive. The cumulative advantages of congruent high resources demonstrate that even employees with moderate to high resource levels can benefit from targeted enhancement. Organizations should implement regular resource profiling assessments to identify incongruence, rather than only intervening after mental health problems emerge [59]. This is particularly critical in the gaming industry, where chronic resource exhaustion creates high risks for mental health issues.

Third, interventions must consider the marital status to maximize effectiveness. For married employees, integrating family support into workplace mental health programs will yield the greatest benefits for anxiety reduction. For separated/divorced employees, dual-resource enhancement programs that build both resilience and alternative social support systems are most effective. For single employees, who showed no significant congruence effect in this study, interventions should move beyond resilience and social support programs to individualized approaches that address their unique social support needs, which may be more diverse and less stable than those of married individuals.

## Limitations

Several limitations should be noted. First, the cross-sectional design cannot establish the temporal direction of resource congruence and mental health outcomes; longitudinal studies are needed to confirm the causal relationships. Second, while the overall sample size was adequate (n = 703), subgroup analyses may be underpowered due to uneven group distributions, particularly for the separated/divorced subgroup. Third, other personal and conditional resources (e.g., personality traits, coping strategies) that may influence the observed effects were not included [60, 61]. Finally, this study did not empirically test the proposed resource alignment intervention framework. Future research should address these limitations to develop more effective and culturally adapted mental health support systems for Macau’s gaming workforce.

## Supplementary Information

Supplementary Material 1.

Supplementary Material 2.

Supplementary Material 3.

## Data Availability

The datasets generated and analyzed during the current study are not publicly available due to privacy protection requirements, but are available from the corresponding author on reasonable request.
