# Supplementary material for: Toward targeted mental health support: the (in)congruence effect of resilience and social support among casino employees in Macau
Source: BMC Public Health. 2026 May 12;26:2059. doi: 10.1186/s12889-026-27684-8 (PMC13340398; doi:10.1186/s12889-026-27684-8)
Supplement: Supplementary file 1 — Supplementary Material 1. [file 12889_2026_27684_MOESM1_ESM.docx]

**Table S1.** Means (SDs) for psychological resilience, social support, and mental health outcomes and difference scores for psychological resilience minus social support for the discrepant groups (PR < or > SS) and the congruent group (PR = SS).

|  | PR < SS (n = 203) | PR = SS (n = 303) | PR > SS (n = 197) | *F* |
| --- | --- | --- | --- | --- |
| Resilience | 14.936  (7.330) | 21.234  (7.856) | 24.056  (10.683) |  |
| PR | -0.567  (0.788) | 0.111  (0.845) | 0.414  (1.148) | 59.915^***^ |
| Social support | 62.798  (11.104) | 55.231  (13.202) | 42.802  (16.033) |  |
| SS | 0.572  (0.716) | 0.084  (0.851) | -0.718  (1.034) | 111.823^***^ |
| Anxiety | 6.532  (5.495) | 6.607  (5.529) | 6.990  (6.234) | 0.379 |
| Depression | 8.695  (5.971) | 8.914  (6.251) | 9.584  (6.869) | 1.081 |
| Perceived stress | 28.877  (6.308) | 26.386  (7.984) | 26.569  (9.001) | 6.881^***^ |
| Difference score | -1.138  (0.640) | 0.027  (0.289) | 1.132  (0.615) | 990.998^***^ |

Note: PR = scaled psychological resilience; SS = scaled social support; Difference score = PR minus SS. **p* < 0.05; ***p* < 0.01; ****p* < 0.001

**Table S2.** Model stability analyses by controlling the covariates.

| Domain | Anxiety  (SE) | | Depression  (SE) | | Perceived stress  (SE) | |
| --- | --- | --- | --- | --- | --- | --- |
|  | Model 1 | Model 2 | Model 1 | Model 2 | Model 1 | Model 2 |
| *b0* | 7.404***  (0.270) | 5.922***  (1.789) | 9.870***  (0.299) | 10.537***  (2.229) | 28.201***  (0.284) | 31.778***  (2.121) |
| *age* |  | -0.163  (0.282) |  | -0.394  (0.317) |  | -0.551  (0.313) |
| *gender* |  | -0.507  (0.438) |  | -0.683  (0.488) |  | -0.487  (0.474) |
| *Working Experience* |  | -0.150  (0.129) |  | -0.267  (0.141) |  | -0.158  (0.143) |
| *Educational Levels* |  | -0.224  (0.175) |  | -0.183  (0.193) |  | -0.114  (0.197) |
| *Marital Status* |  | 0.034  (0.324) |  | -0.653  (0.386) |  | -1.109**  (0.388) |
| *Position* |  | 0.400**  (0.153) |  | 0.324  (0.173) |  | 0.333  (0.172) |
| *Income* |  | -0.052  (0.257) |  | -0.252  (0.300) |  | 0.193  (0.318) |
| *Work Shift* |  | 1.187*  (0.500) |  | 1.922**  (0.624) |  | 0.285  (0.609) |
| *b1*~resilience | -1.002***  (0.253) | -1.028***  (0.252) | -0.924***  (0.272) | -0.911***  (0.270) | -4.074***  (0.283) | -4.074***  (0.282) |
| *b2*~social support | -1.716***  (0.251) | -1.593***  (0.258) | -2.081***  (0.273) | -1.914***  (0.278) | -1.640***  (0.291) | -1.537***  (0.293) |
| *b3*~resilience^2^ | -0.501*  (0.197) | -0.574**  (0.201) | -0.620**  (0.215) | -0.703**  (0.217) | -0.969***  (0.234) | -0.947***  (0.236) |
| *b4*~social support^2^ | -0.325  (0.243) | -0.281  (0.243) | -0.340  (0.234) | -0.281  (0.234) | 0.006  (0.239) | 0.030  (0.239) |
| *b5*~resilience*social support | 0.225  (0.266) | 0.265  (0.262) | 0.252  (0.264) | 0.290  (0.257) | -0.164  (0.283) | -0.184  (0.274) |
| *a1* | -2.717***  (0.285) | -2.620***  (0.286) | -3.006***  (0.324) | -2.824***  (0.322) | -5.714***  (0.335) | -5.611***  (0.328) |
| *a2* | -0.600*  (0.278) | -0.590*  (0.272) | -0.707*  (0.327) | -0.694*  (0.315) | -1.127***  (0.304) | -1.100***  (0.290) |
| *a3* | 0.714  (0.416) | 0.565  (0.423) | 1.157**  (0.438) | 1.003*  (0.443) | -2.434***  (0.466) | -2.537***  (0.473) |
| *a4* | -1.051*  (0.463) | -1.120*  (0.457) | -1.212**  (0.433) | -1.273**  (0.425) | -0.799  (0.485) | -0.733  (0.473) |
| *a5* | -0.176  (0.348) | -0.294  (0.355) | -0.280  (0.353) | -0.422  (0.360) | -0.976**  (0.375) | -0.977*  (0.382) |
| *R^2^* | 0.176 | 0.198 | 0.182 | 0.214 | 0.442 | 0.457 |
| *AIC* | 4324.1 | 4320.0 | 4467.2 | 4456.6 | 4508.3 | 4503.9 |
| *BIC* | 4356.0 | 4383.7 | 4499.1 | 4520.3 | 4540.2 | 4567.6 |

Model 1 did not included the covariates, while Model 2 included the covariates: sex assigned at birth (female, male), age group (21–35, 36–45, 46–55, 56–65 years), working experience (0–5, 5–10, 10–15, 15–20, 20 years and above), educational level (junior secondary education or below, senior secondary education, tertiary education, undergraduate level or higher), marital status (married, not married, separated/divorced), job position (services and sales workers, managers and administrators, technicians and associate professionals, clerks [excluding croupiers], croupiers), monthly income in MOP (0–20,000, 20,001–30,000, 30,001–40,000, 40,001 and above), and work schedule (shift work, fixed-schedule work, Irregular shift).. *p < 0.05; **p < 0.01; ***p < 0.001.

**Table S3.** Model suitability analyses.

| Domain | Anxiety | | | Depression | | | Perceived stress | | |
| --- | --- | --- | --- | --- | --- | --- | --- | --- | --- |
|  | Model 1 | Model 2 | Model 3 | Model 1 | Model 2 | Model 3 | Model 1 | Model 2 | Model 3 |
| *b0* | 5.332**  (1.783) | 5.634**  (1.785) | 5.922***  (1.789) | 9.859***  (2.187) | 10.216***  (2.220) | 10.537***  (2.229) | 30.690***  (2.112) | 31.544***  (2.122) | 31.778***  (2.121) |
| *age* | -0.217  (0.280) | -0.205  (0.280) | -0.163  (0.282) | -0.462  (0.315) | -0.448  (0.315) | -0.394  (0.317) | -0.677*  (0.310) | -0.643*  (0.311) | -0.551  (0.313) |
| *gender* | -0.421  (0.437) | -0.437  (0.436) | -0.507  (0.438) | -0.576  (0.486) | -0.594  (0.485) | -0.683  (0.488) | -0.303  (0.472) | -0.347  (0.472) | -0.487  (0.474) |
| *Working Experience* | -0.149  (0.128) | -0.146  (0.128) | -0.150  (0.129) | -0.264  (0.139) | -0.260  (0.139) | -0.267  (0.141) | -0.148  (0.142) | -0.138  (0.142) | -0.158  (0.143) |
| *Educational Levels* | -0.281  (0.173) | -0.285  (0.173) | -0.224  (0.175) | -0.251  (0.191) | -0.255  (0.191) | -0.183  (0.193) | -0.190  (0.198) | -0.200  (0.197) | -0.114  (0.197) |
| *Marital Status* | -0.023  (0.324) | -0.035  (0.324) | 0.034  (0.324) | -0.721  (0.384) | -0.735  (0.385) | -0.653  (0.386) | -1.168**  (0.387) | -1.201**  (0.387) | -1.109**  (0.388) |
| *Position* | 0.392*  (0.153) | 0.386*  (0.153) | 0.400**  (0.153) | 0.315  (0.173) | 0.307  (0.173) | 0.324  (0.173) | 0.324  (0.172) | 0.306  (0.172) | 0.333  (0.172) |
| *Income* | 0.049  (0.257) | 0.031  (0.256) | -0.052  (0.257) | -0.129  (0.300) | -0.150  (0.299) | -0.252  (0.300) | 0.380  (0.315) | 0.330  (0.315) | 0.193  (0.318) |
| *Work Shift* | 1.183*  (0.491) | 1.132*  (0.498) | 1.187*  (0.500) | 1.912**  (0.613) | 1.852**  (0.622) | 1.922**  (0.624) | 0.323  (0.602) | 0.180  (0.609) | 0.285  (0.609) |
| *b1*~resilience | -1.065***  (0.240) | -1.047***  (0.244) | -1.028***  (0.252) | -0.961***  (0.261) | -0.939***  (0.265) | -0.911***  (0.270) | -4.203***  (0.283) | -4.150***  (0.285) | -4.074***  (0.282) |
| *b2*~social support | -1.483***  (0.263) | -1.551***  (0.268) | -1.593***  (0.258) | -1.797***  (0.278) | -1.877***  (0.279) | -1.914***  (0.278) | -1.391***  (0.281) | -1.582***  (0.291) | -1.537***  (0.293) |
| *b3*~resilience^2^ |  |  | -0.574**  (0.201) |  |  | -0.703**  (0.217) |  |  | -0.947***  (0.236) |
| *b4*~social support^2^ |  |  | -0.281  (0.243) |  |  | -0.281  (0.234) |  |  | 0.030  (0.239) |
| *b5*~resilience*social support |  | -0.232  (0.230) | 0.265  (0.262) |  | -0.274  (0.261) | 0.290  (0.257) |  | -0.655**  (0.246) | -0.184  (0.274) |
| *a1* | -2.548***  (0.284) | -2.598***  (0.277) | -2.620***  (0.286) | -2.759***  (0.319) | -2.817***  (0.313) | -2.824***  (0.322) | -5.594***  (0.322) | -5.733***  (0.324) | -5.611***  (0.328) |
| *a2* |  | -0.232  (0.230) | -0.590*  (0.272) |  | -0.274  (0.261) | -0.694*  (0.315) |  | -0.655**  (0.246) | -1.100***  (0.290) |
| *a3* | 0.418  (0.416) | 0.505  (0.431) | 0.565  (0.423) | 0.836  (0.434) | 0.938*  (0.445) | 1.003*  (0.443) | -2.812***  (0.463) | -2.568***  (0.476) | -2.537***  (0.473) |
| *a4* |  | 0.232  (0.230) | -1.120*  (0.457) |  | 0.274  (0.261) | -1.273**  (0.425) |  | 0.655**  (0.246) | -0.733  (0.473) |
| *a5* |  |  | -0.294  (0.355) |  |  | -0.422  (0.360) |  |  | -0.977*  (0.382) |
| *R^2^* | 0.180 | 0.182 | 0.198 | 0.193 | 0.196 | 0.214 | 0.431 | 0.441 | 0.457 |
| *AIC* | 4330.929 | 4330.868 | 4320.884 | 4466.876 | 4466.510 | 4454.622 | 4330.929 | 4330.868 | 4320.884 |
| *BIC* | 4385.594 | 4390.088 | 4389.214 | 4521.541 | 4525.729 | 4522.952 | 4385.594 | 4390.088 | 4389.214 |

Model 1 only included first-order terms for resilience and social support. Model 2 included the first-order and interactions terms. Model 3 included the full model of RSA analysis.
